# A Geometric Interpretation of Stochastic Gradient Descent Using Diffusion Metrics

**DOI:** 10.3390/e22010101

**Published:** 2020-01-15

**Authors:** Rita Fioresi, Pratik Chaudhari, Stefano Soatto

**Affiliations:** 1 Dipartimento di Matematica, piazza Porta San Donato 5, University of Bologna, 40126 Bologna, Italy; 2 Department of Electrical and Systems Engineering, University of Pennsylvania, Philadelphia, PA 19104, USA; pratikac@seas.upenn.edu; 3 Computer Science Department, University of California, Los Angeles, CA 90095, USA; soatto@cs.ucla.edu

**Keywords:** stochastic gradient descent, deep learning, general relativity

## Abstract

This paper is a step towards developing a geometric understanding of a popular algorithm for training deep neural networks named stochastic gradient descent (SGD). We built upon a recent result which observed that the noise in SGD while training typical networks is highly non-isotropic. That motivated a deterministic model in which the trajectories of our dynamical systems are described via geodesics of a family of metrics arising from a certain diffusion matrix; namely, the covariance of the stochastic gradients in SGD. Our model is analogous to models in general relativity: the role of the electromagnetic field in the latter is played by the gradient of the loss function of a deep network in the former.

## 1. Introduction

Deep neural networks are high-dimensional machine learning models that have demonstrated impressive performance on a number of challenging tasks in computer vision, natural language processing and reinforcement learning [1,2]. These models are typically trained to minimize the misprediction error compared to large amounts of human-annotated data. A large number of diverse models with varying properties are prevalent in these application domains. Despite this diversity, stochastic gradient descent (SGD) is the gold standard for training deep neural networks. It has been shown to obtain good generalization performance; i.e., to train a model that performs well on new data, across a wide range of applications. In spite of this popularity and efficacy, a precise understanding of SGD for deep learning remains elusive.

This paper develops a geometric understanding of stochastic gradient descent. We build upon the work of [3], wherein the authors model the dynamics of SGD as a stochastic differential equation with state-dependent Gaussian noise. We interpret the covariance of this noise, called the diffusion matrix henceforth, as a metric on the parameter space. Our result provides a deterministic Equation (10) that can be compared to SGD near equilibrium points Equation (1). We write the diffusion matrix D(x) in the form Equation (3) to show how it fundamentally captures the anisotropy of the dynamical system underlying SGD. This clarifies how D(x) is one of the key factors that differentiates steady-state solutions of SGD from those of ordinary gradient descent GD (see comparison in [3,4,5]). Using the diffusion matrix, we then define a family of metrics on the parameter space that we call *diffusion metrics*. We then take the Einstein equation describing the geodesic on a Riemannian manifold, for the motion of a particle subject to a gravitational and electromagnetic field. We replace the electromagnetic force by the ordinary gradient while gravity is taken into account using the diffusion metric itself. After some mild hypotheses on the architecture of the neural network, we obtain the result that geodesics with respect to this equation correspond precisely to the evolution of a dynamical system, which is not subject to Euclidean gradient descent but to relativistic gradient descent (RGD) with respect to the family of diffusion metrics.

In the end, we obtain Equation (10), which is along the same vein as natural gradient descent in [6], but whose significance is much deeper in the context of SGD, since it stems from the anisotropy of the gradients with respect to the various parameters. This anisotropy encodes the difference between the dynamics of GD and those of SGD. We also compare our result with the ones in [3] and show them to be perfectly compatible. In the end we provide Appendix A for the reader convenience, with a quick review of some key facts of Riemannian geometry.

## 2. Continuous-Time SGD and the Diffusion Matrix

Stochastic gradient descent performs an update of the weights *x* of a neural network, replacing the ordinary gradient of the loss function $f=\frac{1}{N}\sum_{i=1}^{N}f_{i}$ with $\nabla_{B}f$:

(1) $$dx=-\nabla_{B}fdt,\;\nabla_{B}f=\frac{1}{\left|B\right|}\sum_{i\in B}\nabla f_{i},$$

where dx represents the continuous version of the weight update at step *j*: $x_{j+1}=x_{j}-\eta \nabla_{B}f\left(x_{j}\right)$, with the learning rate η incorporated into the expression of $\nabla_{B}f$, and B is the mini-batch. In the expression of the loss function $f=\frac{1}{N}\sum_{i=1}^{N}f_{i}$, $f_{i}$ is the loss relative to the *i*-th element in our dataset Σ of size |Σ|=N. We assume that weights belong to a compact subset $\Omega \subset R^{d}$ and that the $f_{i}s$ satisfy suitable regularity conditions (see [3] Section 2 for more details).

We define the *diffusion matrix* as the product of the size of the mini-batch |B| and the variance of $\nabla_{B}f$, viewed as a random variable, $\phi :\Sigma ⟶R^{d}$, $\phi \left(z_{i}\right)=\nabla f_{i}$:

(2) $$D\left(x\right)=E[\left(\phi -E\left[\phi \right]\right){\left(\phi -E\left[\phi \right]\right)}^{t}]$$

Notice that $D\left(x\right)\in R^{d\times d}$ and does not depend on the size of the mini-batch; it only depends on the weights *x*, loss function *f* and the dataset Σ. With a direct calculation one can show that:

(3) $$\begin{array}{cc} D & =\frac{1}{N}\sum_{k}\left(\nabla f_{k}\right){\left(\nabla f_{k}\right)}^{t}-\left(\nabla f\right){\left(\nabla f\right)}^{t}=\frac{1}{N^{2}}\left(\langle \partial_{r}\hat{f},\partial_{s}\hat{f}\rangle \right) \end{array}$$

where:

$$\hat{f}=\left(f_{1}-f_{2},f_{1}-f_{3},\cdots ,f_{N-1}-f_{N}\right)\in R^{N(N-1)/2}$$

and 〈,〉 is the euclidean scalar product. In fact:

$$\begin{array}{cc} D_{rs} & =\frac{1}{N}\sum_{k=1}^{N}\partial_{r}f_{k}\;\partial_{s}f_{k}-\frac{1}{N^{2}}\sum_{i,j=1}^{N}\partial_{r}f_{i}\;\partial_{s}f_{j}\\ & =\frac{1}{N^{2}}[N\left(\partial_{r}f_{1}\;\partial_{s}f_{1}+\cdots +\partial_{r}f_{N}\;\partial_{s}f_{N}\right)+\\ & -(\partial_{r}f_{1}\;\partial_{s}f_{1}+\partial_{r}f_{1}\;\partial_{s}f_{2}+\cdots +\partial_{r}f_{N}\;\partial_{s}f_{N})]=\\ & =\frac{1}{N^{2}}[\left(N-1\right)\partial_{r}f_{1}\;\partial_{s}f_{1}-\partial_{r}f_{1}\;\partial_{s}f_{2}-\cdots -\partial_{r}f_{1}\;\partial_{s}f_{N}+\\ & -\partial_{r}f_{2}\;\partial_{s}f_{1}+\left(N-1\right)\partial_{r}f_{2}\;\partial_{s}f_{2}-\cdots -\partial_{r}f_{2}\;\partial_{s}f_{N}+\cdots \\ & -\partial_{r}f_{N}\;\partial_{s}f_{1}-\partial_{r}f_{N}\;\partial_{s}f_{2}+\cdots +\left(N-1\right)\partial_{r}f_{N}\;\partial_{s}f_{N}] \end{array}$$

which gives:

$$\begin{array}{cc} D_{rs} & =\frac{1}{N^{2}}[\left(\partial_{r}f_{1}-\partial_{r}f_{2}\right)\left(\partial_{s}f_{1}-\partial_{s}f_{2}\right)+\left(\partial_{r}f_{1}-\partial_{r}f_{3}\right)\left(\partial_{s}f_{1}-\partial_{s}f_{3}\right)+\cdots \\ & +\left(\partial_{r}f_{1}-\partial_{r}f_{N}\right)\left(\partial_{s}f_{1}-\partial_{s}f_{N}\right)+\left(\partial_{r}f_{2}-\partial_{r}f_{3}\right)\left(\partial_{s}f_{2}-\partial_{s}f_{3}\right)+\cdots \\ & +\left(\partial_{r}f_{N-1}-\partial_{r}f_{N}\right)\left(\partial_{s}f_{N-1}-\partial_{s}f_{N}\right)=\frac{1}{N^{2}}\left(\langle \partial_{r}\hat{f},\partial_{s}\hat{f}\rangle \right). \end{array}$$

The diffusion matrix measures effectively the *anisotropy* of our data: D=0 if and only if $\partial_{r}\left(f_{i}\right)=\partial_{r}\left(f_{j}\right)$ for all r=1,⋯,d and i,j=1,⋯,N. In other words, the diffusion matrix measures how the loss of each datum depends, at first order, on the weights in a different way with respect to the loss of each of the other data-points. So, it tells us how much we should expect the SGD dynamics to differ from the GD one. Notice that the expression (Equation (3)) gives us immediately a bound on the rank of *D*; namely, rk(D)≤N−1.

The Table 1 suggests that in many algorithms currently available, the diffusion matrix has low rank; hence, it is singular; this just by comparing the size *d* of *D* and its rank which bound by *N*. This fact turns out to be very important in the construction of the diffusion metrics, as we will see below.

## 3. Diffusion Metrics and General Relativity

The evolution of a dynamical system in general relativity takes place along the geodesics according to the metric imposed on the Minkowski space by the presence of gravitational masses. The equation for such geodesics, once Einstein’s equation is solved, is:

(4) $$\frac{d^{2}x^{\mu }}{dt^{2}}\;+\;\Gamma_{\rho \sigma }^{\mu }\frac{dx^{\rho }}{dt}\frac{dx^{\sigma }}{dt}=\frac{q}{m}F_{\nu }^{\mu }\frac{dx^{\nu }}{dt},$$

where $\Gamma_{\rho \sigma }^{\mu }$ are the Christoffel symbols for the Levi–Civita connection:

(5) $$\Gamma_{uv}^{w}=\frac{1}{2}g^{wz}\left(\partial_{u}g_{vz}-\partial_{z}g_{uv}+\partial_{v}g_{uz}\right)$$

and $\frac{q}{m}F_{\nu }^{\mu }$ is a term regarding an external force; e.g., one coming from an electromagnetic field.

If we take the time derivative of the differential equation underlying the ordinary (i.e., non stochastic) gradient descent:

$$\frac{d^{2}x^{\mu }}{dt^{2}}\;=-\frac{d}{dt}\;\partial_{\mu }f$$

and compare with Equation (4), observe that $-\frac{d}{dt}\;\partial_{\mu }f$ effectively replaces the force term $\left(q/m\right)F_{\nu }^{\mu }\frac{dx^{\nu }}{dt}$. Hence, the geodesic equation Equation (4) models the ordinary GD equation if we take a constant metric and replace the force term with the gradient of the loss; furthermore, this corresponds to the condition D=0 in SGD dynamics Equation (1).

This suggests that one may define a metric dependent on the diffusion matrix; this metric should become constant when D=0. As a side remark, notice that since *D* is singular in many important practical applications (see Table 1), it is not reasonable to use it to define the metric itself. On the other hand, since *D* measures the anisotropy of the weight space, it is reasonable to employ it to perturb the euclidean metric. So the stochastic nature of the dynamical system ruled by the SGD is replaced by a perturbation of the dynamics for the ordinary gradient descent. As an analogy, the presence of (small) masses in space, in the weak field approximation (see [7]) of general relativity, generates gravity; this motivates our (small) deformation of the euclidean metric using the diffusion matrix.

At each point x∈Ω, we define a metric called *diffusion metric* as

(6) g(x)=id+ε(x)D(x)

with $\varepsilon (x)<1/M_{x}$, where $M_{x}=\max\left\{\lambda_{k}:\;\lambda_{k}\;\mathrm{is}\;\mathrm{an}\;\mathrm{eigenvalue}\;\mathrm{of}\;D\left(x\right)\right\}$. This ensures that *g* is non-singular at each x∈Ω. We have, thus, defined a family of metrics that depend on the real parameter ε. We expect this model to approximate the solution to the Fokker–Planck equation when the parameter $\beta^{-1}$, whose interpretation is related with the temperature, is very small (see [3] for the notation and more details).

Notice that our heuristic hypothesis on ε allows us to make the so called *weak field approximation* (see [7]):

$$g^{-1}=\mathrm{id}-\varepsilon D\left(x\right).$$

Hence, we have the following expression for the Christoffel’s symbols (in this approximation we discard $O(\varepsilon^{2})$):

(7) $$\Gamma_{uv}^{w}=\frac{1}{2}\sum_{z}\left(\delta_{wz}-\varepsilon_{wz}^{d}\right)\;\varepsilon^{\;}\left(\partial_{u}d_{vz}-\partial_{z}d_{uv}+\partial_{v}d_{uz}\right)=\frac{\varepsilon }{2}\left.(\partial_{u}d_{vw}-\partial_{w}d_{uv}+\partial_{v}d_{uw}\right)$$

where *d_ij_* are the coefficients of *D*, and *δ_wz_* is the Kronecker delta.

Let us now compute the Christoffel’s symbols and then substitute them into the geodesic equation given by Equation (4).

$$\begin{array}{cc} \Gamma_{ij}^{k} & =\frac{\varepsilon }{2N^{2}}[\langle \partial_{i}\partial_{j}\hat{f},\partial_{k}\hat{f}\rangle +\langle \partial_{j}\hat{f},\partial_{i}\partial_{k}\hat{f}\rangle -\langle \partial_{i}\partial_{k}\hat{f},\partial_{j}\hat{f}\rangle -\langle \partial_{i}\hat{f},\partial_{k}\partial_{j}\hat{f}\rangle \\ & +\langle \partial_{j}\partial_{k}\hat{f},\partial_{i}\hat{f}\rangle +\langle \partial_{k}\hat{f},\partial_{i}\partial_{j}\hat{f}\rangle ]=\frac{\varepsilon }{N^{2}}\langle \partial_{i}\partial_{j}\hat{f},\partial_{k}\hat{f}\rangle . \end{array}$$

Let us substitute in Equation (4) (writing the sum now):

(8) $$\frac{d^{2}x^{k}}{dt^{2}}\;+\;\frac{\varepsilon }{N^{2}}\sum_{i,j}\langle \partial_{i}\partial_{j}\hat{f},\partial_{k}\hat{f}\rangle \frac{dx^{i}}{dt}\frac{dx^{j}}{dt}=\frac{d}{dt}\partial_{k}f.$$

Let us concentrate on the expression:

$$\begin{array}{cc} \frac{\varepsilon }{N^{2}}\sum_{i,j}\langle \partial_{i}\partial_{j}\hat{f},\partial_{k}\hat{f}\rangle \frac{dx^{i}}{dt}\frac{dx^{j}}{dt} & =\frac{\varepsilon }{N^{2}}\sum_{i,j,\alpha }\partial_{i}\partial_{j}{\hat{f}}_{\alpha }\;\partial_{k}{\hat{f}}_{\alpha }\frac{dx^{i}}{dt}\frac{dx^{j}}{dt}\\ & =\frac{\varepsilon }{N^{2}}\sum_{\alpha }\frac{d^{2}{\hat{f}}_{\alpha }}{dt^{2}}\partial_{k}{\hat{f}}_{\alpha }. \end{array}$$

Now, we take the integral in *dt* (we compute the parts twice):

$$\begin{array}{cc} \frac{\varepsilon }{N^{2}}\int \sum_{\alpha }\frac{d^{2}{\hat{f}}_{\alpha }}{dt^{2}}\partial_{k}{\hat{f}}_{\alpha }\;dt & =\frac{\varepsilon }{N^{2}}\sum_{\alpha }\left[\frac{d{\hat{f}}_{\alpha }}{dt}\partial_{k}{\hat{f}}_{\alpha }-\int \frac{d{\hat{f}}_{\alpha }}{dt}\frac{\partial_{k}{\hat{f}}_{\alpha }}{dt}\;dt\right]\\ & =\frac{\varepsilon }{N^{2}}\sum_{\alpha }\left[\frac{d{\hat{f}}_{\alpha }}{dt}\partial_{k}{\hat{f}}_{\alpha }-{\hat{f}}_{\alpha }\frac{d}{dt}\partial_{k}{\hat{f}}_{\alpha }+\int {\hat{f}}_{\alpha }\frac{d^{2}}{dt^{2}}\partial_{k}{\hat{f}}_{\alpha }\;dt\right]. \end{array}$$

Notice that, in many practical applications we have:

(9) $$\frac{d^{2}}{dt^{2}}\partial_{k}{\hat{f}}_{\alpha }=0$$

because $\partial_{i}\partial_{j}\partial_{k}\hat{f}=0.$

Hence

$$\begin{array}{cc} \frac{\varepsilon }{N^{2}}\int \sum_{\alpha }\frac{d^{2}{\hat{f}}_{\alpha }}{dt^{2}}\partial_{k}{\hat{f}}_{\alpha }\;dt & =\frac{\varepsilon }{N^{2}}\sum_{\alpha ,\ell }\left[\partial_{\ell }{\hat{f}}_{\alpha }\frac{dx^{\ell }}{dt}\partial_{k}{\hat{f}}_{\alpha }-{\hat{f}}_{\alpha }\frac{d}{dt}\partial_{k}{\hat{f}}_{\alpha }\right]\\ & =\varepsilon \sum_{\ell }d_{k,\ell }\frac{dx^{\ell }}{dt}-\frac{\varepsilon }{N^{2}}\sum_{\alpha }{\hat{f}}_{\alpha }\frac{d}{dt}\partial_{k}{\hat{f}}_{\alpha }. \end{array}$$

We now substitute the obtained expression into the Equation (8), taking the integral in *dt*:

$$\frac{dx^{k}}{dt}\;+\varepsilon \sum_{\ell }d_{k,\ell }\frac{dx^{\ell }}{dt}-\frac{\varepsilon }{N^{2}}\sum_{\alpha }{\hat{f}}_{\alpha }\frac{d}{dt}\partial_{k}{\hat{f}}_{\alpha }=-\partial_{k}f.$$

We may assume $\frac{\varepsilon }{N^{2}}$ very small, as motivated by Equation (1); hence, discard this term. Writing the equation into vector form, we have:

$$\frac{dx}{dt}+\varepsilon D\frac{dx}{dt}=-\nabla f.$$

By the weak field approximation ${\left(I+\varepsilon D\right)}^{-1}≅\left(I-\varepsilon D\right)$, we can write:

(10) $$\frac{dx}{dt}=-\left(I-\varepsilon^{D}\right)\nabla f=-\nabla_{D}f,$$

where $\nabla_{D}f$ is the gradient computed according to the diffusion metric Equation (6).

We can summarize our result as follows: *the SGD equation* Equation (1) can be replaced, provided the approximation Equation (9) holds, by the deterministic equation Equation (10), where the dynamical system evolves with respect to the gradient computed according to the diffusion metric Equation (6).

We now want to compare our result Equation (10) with [3] Section 1, in order to understand how the steady state solutions of Equation (10) compare to the SGD steady state solutions described by Equation (1). In [3], the authors regard SGD as minimizing the function Φ instead of our loss *f*. Let us focus on (8) in [3], where the relation between *f* and Φ is discussed. In our case, we take ∇Φ = ∇*_D_f* so that Equation (11) becomes

(11) $$-\nabla f\left(x\right)+\tilde{D}\left(x\right)\nabla \Phi \left(x\right)=0,$$

where $\tilde{D}\left(x\right)=\left(I+\varepsilon D\left(x\right)\right)$ is the diffusion metric. If the term *D(x)* in (8) in [3] is spelled out as our $\tilde{D}\left(x\right)$, we can write such equation as:

(12) $$j\left(x\right)=-\nabla f\left(x\right)+\tilde{D}\left(x\right)\nabla \Phi \left(x\right)-\beta^{-1}\nabla \cdot \tilde{D}\left(x\right).$$

Notice that according to our approximation Equation (9), $\nabla \cdot \tilde{D}\left(x\right)=0$. Hence, Equation (12) (that is (8) in [3]) is perfectly compatible with our treatment, and furthermore, the assumption 4 in [3] is fully justified by the fact that *j(x)* = 0.

## 4. Conclusions

The general relativity (GR) model helps to provide a deterministic approach the evolution of the dynamical system described by SGD, through the use of the diffusion metric accounting for the anisotropy of the system. Our results are compatible with [3]; moreover, they give GD for an isotropic system. We plan to explore deep neural networks mixing our GR model with energy models in a forthcoming paper (see also [9]). This will provide new geometric insight to the theory.

## Figures and Tables

**Table 1:** Values for *N* and *d* for various architectures on CIFAR and SVHN datasets (see [8]).

Architecture	d=|Weights|	N=|Data|, CIFAR	N=|Data|, SVHN
ResNet	1.7 M	60 K	600 K
Wide ResNet	11 M	60 K	600 K
DenseNet (k = 12)	1 M	60 K	600 K
DenseNet (k = 24)	27.2 M	60 K	600 K

## Appendix A. Riemannian Geometry

We collected a few well known facts of Riemannian geometry, and invite the reader to consult [10] for more details.

In Riemannian geometry, we define a metric *g* on a smooth manifold *M*, as a smooth assignment $p\mapsto g_{p}$, which gives, for each p∈M, a (non degenerate) scalar product on $T_{p}\left(M\right)$, the tangent space of *M* at *p*. Usually, this scalar product is assumed to be a positive definite; however, for general relativity, it is necessary to drop this assumption, so that we speak of a *pseudometric*, because our main example is the Minkowski metric. To ease the terminology, we say "metric" to include this more general setting as well.

Once a metric is given, we say that *M* is a *Riemannian manifold*. In local coordinates, $x^{1},\ldots ,x^{n}$, we express the metric using 1-forms:

$$g=\sum_{i,j}g_{ij}\;dx^{i}⊗dx^{j}.$$

where

$$g_{ij}{|}_{p}:=g_{p}\left({\left.\frac{\partial }{\partial x^{i}}\right|}_{p},{\left.\frac{\partial }{\partial x^{j}}\right|}_{p}\right)\;\mathrm{and}\;\left\{\frac{\partial }{\partial x^{1}}{{|}_{p},\cdots ,\frac{\partial }{\partial x^{n}}|}_{p}\right\}$$

is a basis of the tangent space $T_{p}M$.

For example $R^{n}$, identified with its tangent space at every point, has a canonical or standard metric given by:

$$g_{p}^{\mathrm{can}}:T_{p}R^{n}\times T_{p}R^{n}⟶R,\;\left(\sum_{i}a_{i}\frac{\partial }{\partial x^{i}},\sum_{j}b_{j}\frac{\partial }{\partial x^{j}}\right)⟼\sum_{i}a_{i}b_{i}.$$

Here, $g_{ij}^{\mathrm{can}}=\delta_{ij}$.

Usually, we drop the ∑ symbol, following Einstein’s convention.

An *affine connection* ∇ on a smooth manifold *M* is an bilinear map $\left(X,Y\right)\mapsto \nabla_{X}Y$ associating with a pair of vector fields X,Y on *M* another vector field $\nabla_{X}Y$, satisfying:

1. $\nabla_{fX}Y=f\nabla_{X}Y$ for all functions *f* on *M*;
2. $\nabla_{X}\left(fY\right)=df\left(X\right)Y+f\nabla_{X}Y$.

Once this definition is given, it is possible to define ∇ on tensors of every order.

On a Riemannian manifold we have a unique affine connection, the *Levi*–*Civita connection* ∇, which is torsion free and preserves the metric; i.e., ∇g=0. In local coordinates the components of the connection are called the *Christoffel symbols*:

$$\nabla_{\frac{\partial }{\partial x^{i}}}\frac{\partial }{\partial x^{j}}=\Gamma_{ij}^{k}\frac{\partial }{\partial x^{k}}.$$

By the above -mentioned uniqueness, the $\Gamma_{ij}^{k}$s are expressed in terms of the metric components $g_{ij}$:

$$\Gamma_{jk}^{l}=\frac{1}{2}g^{lr}\left(\partial_{k}g_{rj}+\partial_{j}g_{rk}-\partial_{r}g_{jk}\right),$$

where as usual, $g^{ij}$ are the coefficients of the dual metric tensor; i.e., the entries of the inverse of the matrix $\left(g_{kl}\right)$. The torsion freeness is equivalent to the symmetry

$$\Gamma_{jk}^{l}=\Gamma_{kj}^{l}.$$

In $R^{n}$, the *gradient* of a scalar function *f* is the vector field characterized by the property: $\mathrm{grad}\left(f\right)\cdot v=D_{v}$ (we shall denote it with ∇(f) so that no confusion arises). In other words, its scalar product (in the euclidean metric) with a tangent vector *v* gives the directional derivative of *f* along *v*. When *M* is a Riemannian manifold with metric *g*, the gradient of a function *f* on *M* is defined in the same way, except that the scalar product is now given by *g*. So, in local coordinates, we have:

$$\nabla_{g}f=\frac{\partial f}{\partial x^{i}}g^{ij}\frac{\partial }{\partial x^{j}}.$$

Notice that when $g_{ij}=\delta_{ij}$, we retrieve the usual definition in $R^{n}$.

We end our short summary of the key concepts, with the notion of *geodesic*.

A geodesic γ on a smooth manifold *M* with an affine connection ∇ is a curve defined by the following equation:

$$\nabla_{\dot{\gamma }}\dot{\gamma }=0$$

Geometrically, this expresses the fact that the parallel transport, given by ∇, along the curve γ preserves any tangent vector to the curve. In local coordinates this becomes:

$$\frac{d^{2}\gamma^{\lambda }}{dt^{2}}+\Gamma_{\mu \nu }^{\lambda }\frac{d\gamma^{\mu }}{dt}\frac{d\gamma^{\nu }}{dt}=0\;.$$

Notice that when the metric is constant, we have the familiar equation:

$$\frac{d^{2}\gamma^{\lambda }}{dt^{2}}=0;$$

that is, the geodesics are straight lines.
